# Exploring the Prevalence and Symptom Burden of Small Fiber Neuropathy in Patients With Diabetes Using the Small Fiber Neuropathy Symptoms Inventory Questionnaire (SFN-SIQ)

**DOI:** 10.7759/cureus.93548

**Published:** 2025-09-30

**Authors:** Yashar Mashayekhi, Chinenye Iguh, Sara Baba-Aissa, Mishal Iqbal, Tejashree Nidiginti, Rabia Jalali, Ali A Kashmoola, Mutaz M Abualhab, Racha Al Niazi, Ayaan Rafiq Shaikh, Jerin Xavier Polackal, Ramsha Zahid

**Affiliations:** 1 Medicine, University Hospitals of Leicester NHS Trust, Leicester, GBR; 2 Medicine, Windsor University School of Medicine, Cayon, KNA; 3 General Internal Medicine, Leicester Royal Infirmary, Leicester, GBR; 4 Medicine, Army Medical College, Islamabad, PAK; 5 General Internal Medicine, Calderdale and Huddersfield NHS Foundation Trust, Huddersfield, GBR; 6 Internal Medicine, Jinnah Medical and Dental College, Karachi, PAK; 7 Otolaryngology - Head and Neck Surgery, University of Sharjah, College of Medicine, Sharjah, ARE; 8 Orthodontics, Dubai Health, Dubai, ARE; 9 Internal Medicine, New Vision University, Tbilisi, GEO; 10 Internal Medicine, Combined Military Hospital, Quetta, PAK

**Keywords:** diabetes, neuropathic pain, risk factors, sfn-siq, small fiber neuropathy

## Abstract

Background: Small fiber neuropathy (SFN) is a widely encountered and underdiagnosed complication of diabetes, which is linked with severe agony and loss of quality of life due to the proximity of neuropathic pain, autonomic dysfunction, and sensory problems. Despite its established judgments as a complication, there is limited literature on its consequences and impact on symptoms in patients with diabetes, especially in Pakistan. This research aims to identify the degree of SFN and its burden on symptoms among individuals with diabetes through the Small Fiber Neuropathy Symptoms Inventory Questionnaire (SFN-SIQ).

Methods: This cross-sectional study was conducted between January and June 2025, involving a sample population of 300 patients with diabetes from the outpatient diabetes and endocrinology units in the Combined Military Hospital (CMH) Quetta. The SFN-SIQ, together with the Douleur Neuropathique Questionnaire (DN4), was used to determine neuropathic pain, along with incorporating demographic information into a structured questionnaire. The descriptive statistics, Spearman correlation, and non-parametric testing were used in the analysis of the data.

Results: The study population mostly comprised men (266 men, 89%), with a median age of 60 years or older. The results found significant positive correlations between SFN-SIQ and diabetes-related measures such as duration, type of diabetes, and complications (r = 0.26-0.29). It was observed that females had a greater burden of SFN symptoms compared to males (p = 0.004). Symptoms of SFN are highly related to smoking (p = 0.003), family history of diabetes (p = < 0.001), and the existence of diabetes-related complications (p = 0.001). Type of diabetes, duration, treatment, complications, smoking, and family history were strong predictors of SFN symptom burden, found in the regression analysis (p < 0.01).

Conclusion: In this study, a substantial burden of SFN symptoms among patients with diabetes in Pakistan was found, and there were essential relationships between both symptom severity and diabetes-related factors. The results confirm that early diagnosis and treatment of SFN are crucial in enhancing patients' quality of life and minimizing the long-term effects of diabetes. Regular screening of SFNs in diabetes care centers can contribute to early detection and better treatment of symptoms.

## Introduction

Diabetes mellitus is a chronic metabolic disorder that develops as a result of a hyperglycemic state and results in complications in numerous organs. Its global impact is growing fast to the extent that it is expected to rise to 552 million cases in 2030 [1]. Diabetic neuropathy is among the most prevalent and disabling complications, specifically distal symmetric polyneuropathy, which is commonplace in more than 50% of individuals with diabetes, and it causes immense pain and disability [2].

Small fiber neuropathy (SFN) is a heterogeneous disorder that is mainly associated with diabetes, idiopathic and autoimmune disorders, as well as sodium channel mutations. It has burning pain and autonomic symptoms, and the disease is characterized by a skin biopsy or quantitative sensory testing (QST), although most of the patients have no definite etiology [3,4]. SFN results from the damage of A-delta and C-fibers and is typically marked by neuropathic pain, altered sensation quality, autonomic dysfunction, fatigue, and mood abnormalities. It can be caused by metabolic, toxic, immune, or genetic factors, and the diagnosis is commonly based on skin biopsy rather than standard neurological tests. Management is tailored and multimodal, addressing symptomatic, pathophysiologic, or causal aspects, based on the proven etiology [5,6].

SFN contributes significantly to poor quality of life, especially since autonomic disturbance and chronic pain are involved [7]. Both physical functioning and pain areas are found to be particularly impaired, as the severity of pain and autonomic symptoms (including disturbed sweating and dry mouth) partially predict the reduction in quality of life, regardless of age [8].

Advancements in diagnostic approaches have led to a greater recognition of SFN, with approximately one in three cases being idiopathic. These novel relationships to a range of disorders underscore the importance of clinical scrutiny and early detection and may provide insights into SFN pathophysiology and extra-neuronal systemic effects [9]. The Small Fiber Neuropathy Symptoms Inventory Questionnaire (SFN-SIQ) is a beneficial screening tool used in general clinics, with high sensitivity (86%) and specificity (70%) in pure SFN diagnosis. It helps to identify patients who may require further diagnostics, such as QST or skin biopsy [10].

By critically evaluating the presence and severity of symptoms of diabetic neuropathy in patients with diabetes through the SFN-SIQ, this study aims to increase awareness of a relatively understudied phenomenon and promote early detection, ultimately leading to improved patient outcomes and reduced losses.

Rationale

SFN is a known yet highly unpleasant complication of diabetes. As compared to a significant fiber neuropathy, which has the highest prevalence and is the most commonly screened, SFN pertains to small nerve fiber, which touches on pain, temperature, and autonomic disorders, resulting in various uncomfortable and disabling symptoms. Despite the high occurrence of the condition, a large number of undiagnosed patients exist because the signs are somewhat hidden, and/or awareness is very low among people who work in health-related fields. This highlights the importance of determining the prevalence of SFN among patients with diabetes and the precise burden of individual symptoms in this population.

The SFN-SIQ is a well-designed measure for assessing symptoms of SFN in diabetic populations and the severity of their acuteness. By establishing the level and pattern of frequency of such symptoms in patients, this study can highlight the importance of early detection, in addition to the need for consistent screening. A better understanding of the burden of the symptoms is also likely to lead to more targeted intervention, as well as to better overall quality of life for patients with diabetes with neuropathic complications.

Primary objectives

To determine the prevalence of probable SFN in patients with diabetes using the SFN-SIQ and to assess the prevalence of neuropathic pain using the Douleur Neuropathique Questionnaire (DN4).

Secondary objectives

To evaluate the symptom burden of SFN based on total SFN-SIQ scores; to compare the SFN symptom burden between patients with type 1 and type 2 diabetes; and to examine associations between SFN symptom burden and diabetes-related factors, including duration, treatment, complications, smoking, and family history.

## Materials and methods

Research design

A cross-sectional study design was employed to investigate the prevalence and burden of symptoms associated with SFN in patients with diabetes. Patients were sampled in the outpatient division of the diabetes and endocrinology department at the Combined Military Hospital (CMH) Quetta, providing a wide variety of socioeconomic and cultural backgrounds.

A structured questionnaire was administered to collect data that evaluated the symptoms commonly experienced with SFN and neuropathic pain. Trained research assistants approached patients with diabetes during their routine visits to the department. The aim and methodology of the study were sufficiently explained before participation, and informed consent was obtained. The questionnaire was self-administered by the participants, depending on their literacy capacity, or with the help of a research team member, as is the case. The methodology facilitated the collection of reliable data and ensured courteous and culturally sensitive communication within the local care environment.

Sampling strategy and population size

The sample size was determined using the World Health Organization (WHO) formula to estimate a population proportion with a 95% confidence interval, a 5% margin of error, and an assumed population proportion of 0.5, aiming to achieve maximum variability [11]. According to these parameters, the minimum sample size calculated was approximately 384 respondents.

A total of 385 people were approached during routine outpatient visits to achieve this target, compensating for possible non-responses and incomplete data. Out of this, 336 participants responded, agreeing to take part and complete the questionnaire, which yields a response rate of approximately 87%. Nonetheless, 36 answers were excluded from the final analysis due to the incompleteness of the responses or refusal to continue the data collection process. A total of 300 patients with diabetes with complete data and suitable for analysis were retrieved as the final sample.

A non-random convenience sampling approach was also used, and all participants were invited as they sought check-ups in diabetes and endocrinology units at both government and private hospitals. Although this is slightly less than the ideal calculated sample size, the final sample size of 300 is sufficient to make a meaningful statistical comparison and to conduct subgroup analyses within the diabetic population.

Inclusion and exclusion criteria

Adults aged 18 years and over were included in the study with a diagnosis that was confirmed, either type 1 or type 2 diabetes mellitus. Only individuals who visited outpatient departments of the CMH Quetta to receive routine diabetes care were considered eligible. All individuals who could answer the questionnaire independently or with some assistance and who provided informed consent participated in the study.

Patients who had other known causes of neuropathy other than diabetes, recognized as diabetic neuropathy, including vitamin B12 deficiency, hypothyroidism, chronic kidney disease, or a history of chemotherapy, were excluded. Also, people having neurological conditions that were not associated with diabetes (like multiple sclerosis or Parkinson's disease) and people having psychiatric diseases or conditions that would affect the reporting of symptoms most accurately were dropped. Patients admitted or in severe conditions, such as patients with diabetes, and those who refused before taking the data or gave incomplete data, were also not considered in the final analysis.

Data collection tools

To gather relevant and comprehensive data for this study, a structured questionnaire has been prepared, which will entail three main stages: demographic data, minor fiber neuropathy symptoms, and indicators of neuropathic pain (Appendix 1). The primary objective of the questionnaire was to estimate the prevalence and burden of symptoms related to SFN in patients with diabetes and to identify possible relationships between these symptoms and demographic and clinical characteristics. All the standardized instruments were in the original English language. Therefore, the participants were in a position to respond to the question correctly without making any linguistic or cultural adaptation.

Demographic information

The primary section of this questionnaire was designed to gather extensive information regarding whether they had any complications of diabetes (e.g., neuropathy, retinopathy, nephropathy, or cardiovascular diseases), whether their family had a history of diabetes, and whether they were smoking at the same time (Appendix 1). They were considered to be a demographic and clinical history, allowing for the analysis of subgroups and the determination of the relationship between these factors and the symptom burden of SFN. Some of the variables in this section included age, gender, marital status, level of education, urban or rural residence, and occupation. The clinical information reported included the type of diabetes (type 1 or type 2), the number of years since diabetes diagnosis, and/or the specific treatment plan (e.g., insulin use, oral medications, combination therapy, and so forth). To provide more comprehensive clinical and lifestyle-related information, the study participants were assessed to identify potential risk factors or trends that may signify the presence and severity of minor fiber neuropathy symptoms in patients with diabetes.

Small Fiber Neuropathy Symptoms Inventory Questionnaire (SFN-SIQ)

The second part of the questionnaire entailed the identification and evaluation of symptoms suggestive of SFN. This section included a self-report screening tool, the SFN-SIQ, designed by Brouwer et al. in 2015. It was structured in a manner that quantified the prevalence and severity of sensory and autonomic symptoms, which are hallmarks of SFN, particularly in clinical conditions where diabetes leads to this condition. The SFN-SIQ consists of 13 questions on a binary scale, with an overall score ranging from zero to 13. At any particular instance, the scores zero (absence) and one (presence) are used. The score indicates increased scores when there is an increased intensity of minor fiber-related symptoms. For the SFN-SIQ, no universally accepted diagnostic cutoff exists; instead, higher scores reflect greater symptom burden. The internal consistency of the instrument has been demonstrated to be acceptable, as evidenced by a Cronbach's alpha of approximately 0.75 [12]. The SFN-SIQ was licensed under a User License Agreement with Maastricht University (MHeNS Research Institute), legally represented by Prof. C. Faber.

Douleur Neuropathique Questionnaire (DN4)

The third question aimed to evaluate the occurrence of neuropathic pain because it is common alongside SFN in people with diabetes. In this section of the questionnaire, the DN4 was used; this is a validated diagnostic instrument created by Bouhassira et al. (2005) to diagnose pain. DN4 was specifically developed to aid in differentiating between neuropathic and non-neuropathic pain based on clinical presentation and physical examination results.

The DN4 is a 10-item questionnaire with two parts: seven items are symptom-related (examples of questions are burning, painful cold, electric shocks, tingling, pins and needles, numbness, and itching), and three items are related to physical examination (hypoesthesia to touch, hypoesthesia to pinprick, and pain upon brushing). The items are rated one (yes) or zero (no), and the total score produces a possible range of zero to 10. The DN4 has also demonstrated good psychometric properties, with a Cronbach's alpha value of approximately 0.76, indicating good internal consistency. In the study mentioned, only the interview portion (the first seven items) was applied since it was feasible for use in outpatient settings. Previous validation studies of the DN4-interview, including one in diabetic populations, have demonstrated high diagnostic accuracy with cut-offs of either ≥3 or ≥4; we retained ≥4 to ensure comparability with the wider DN4 literature and to maintain consistency across studies [13,14]. Permission to use the DN4 was obtained prior to data collection. Authorization to use the DN4 was obtained from the Mapi Research Trust under License No. 120562 (issued on 13 August 2025).

Procedure

The participants were recruited from the outpatient departments of endocrinology and diabetes at CMH Quetta. The period during which data was collected lasted four months (January to June 2025). The participants were selected during their regular department visits. They were briefed on the purpose and nature of the study in their preferred language to ensure they understood the information. Before any data were collected, informed consent was obtained from all the participants.

The participants who consented to take part in the study were presented with two options for completing the questionnaire: either face-to-face with the help of specialized research assistants in an environment where the questionnaire was given, or by reading and responding at their own pace. This was to accommodate varying levels of literacy and preferences. A great emphasis was placed on privacy and confidentiality during the data collection process; the names of the participants or any other obvious identifiers were not recorded, and all answers were considered with anonymity in mind. This highly flexible and sensitive process enabled active interaction with a highly differentiated community of patients with diabetes, without compromising ethical interests or cultural sensitivity in the implementation of the research.

Analytical approach

The data were analyzed using IBM SPSS Statistics for Windows, Version 26 (Released 2018; IBM Corp., Armonk, New York, United States). The descriptive statistics of demographic and clinical aspects of the sample population of 300 respondents were summarized in terms of frequencies and percentages. Normality of SFN-SIQ and DN4 scores was assessed with Q-Q plots, which indicated mild deviations from normality. Therefore, non-parametric tests were applied for bivariate analyses. Correlations between variables related to diabetes and scores on neuropathic symptoms were analyzed with Spearman correlation analysis. Comparison of gender was conducted via the Mann-Whitney U test, whereas the Kruskal-Wallis H test was used to compare the neuropathy score based on type of diabetes and type of treatment. Multiple linear regression analyses were conducted using the raw SFN-SIQ and DN4 scores as dependent variables. Predictors included the type and duration of diabetes, treatment, complications, smoking status, and family history. Although the SFN-SIQ and DN4 are bounded scales, they were analyzed as continuous outcomes, which is standard practice in clinical research; the relatively large sample size (N = 300) provides robustness against mild deviations from normality. Additionally, chi-square tests were applied to determine the relationships between age groups, type of diabetes, and family history. All statistical tests were two-sided, and a significance level of p < 0.05 was adopted in the analysis process to establish significant relationships and predictors of SFN in patients with diabetes.

Ethical protocols

The study was conducted in accordance with the ethical principles governing research involving human subjects. Combined Military Hospital (CMH QTA- IERB/111/2025) reviewed and approved the research protocol for the study. The fundamental principles of ethics, such as respect for persons, beneficence, and confidentiality, were adhered to at all costs during the research. Before any data were collected, each participant was thoroughly informed about the study's objectives, procedures, risks, and potential benefits in their preferred language. The recruitment was based on total free will, and written or verbal consent was taken before people were enrolled. It was explicitly stated to participants that they could withdraw from the study at any time without any repercussions or adverse effects on their clinical care. No personally identifiable data were entered; to guarantee confidentiality and save the identity of participants, all data were anonymized at the moment of entry, handling, and analysis. The administered questionnaires were considered complete, and missing data responses on essential items, especially in the standardized symptom inventories, were excluded from the final analysis to maintain the integrity and reliability of the results. The protection, dignity, and privacy of all participants were preserved through adherence to ethical standards throughout.

## Results

Table 1 shows the demographic characteristics of the study sample (N = 300). Most of them were older than 60 years (n = 110, 37%), followed by those between the ages of 51 and 60 years (n = 80, 27%), and then those between 41 and 50 years (n = 50, 17%). The majority of participants were male (n = 266, 89%), and the majority were married (n = 175, 58%), with a significant percentage being divorced/widowed (n = 80, 27%). In terms of education, the most critical proportions were graduates and postgraduates (n = 85, 28% each), with intermediate education also reported by many (n = 60, 20%). Regarding occupation, the participants consisted of a majority of employed individuals (n = 115, 38%) and another large group of retired individuals (n = 90, 30%). Most of them were urban residents (n = 167, 56%), and type 2 diabetes (n = 136, 45%) was more frequent than type 1 (n = 102, 34%), with some of the participants not aware of their kind (n = 62, 21%). The duration of diabetes was mainly reported as one to five years (n = 108, 36%), with common treatments including insulin injections (n = 114, 38%) and oral medications (n = 100, 33%). Diabetic retinopathy (n = 131, 44%) and nephropathy (n = 120, 40%) were the most common complications, with diabetic foot/ulcers (n = 32, 11%) and hypertension (n = 12, 4%) being less prevalent. A high percentage of the total sample were current smokers (n = 214, 71%), and most of the participants had a family history of diabetes (n = 220, 73%).

**Table 1:** Demographic characteristics of participants (N = 300) N: number of participants; f: frequency; %: percentage

Variable	f	%
Age	-	-
18–30 years	25	8
31–40 years	35	12
41–50 years	50	17
51–60 years	80	27
Above 60 years	110	37
Gender	-	-
Male	266	89
Female	34	11
Marital status	-	-
Single	45	15
Married	175	58
Divorced/Widowed	80	27
Educational level	-	-
No formal education	10	3
Primary	20	7
Secondary	40	13
Intermediate	60	20
Graduate	85	28
Postgraduate	85	28
Occupation	-	-
Student	30	10
Unemployed	65	22
Employed	115	38
Retired	90	30
Residence area	-	-
Urban	167	56
Rural	133	44
Type of diabetes	-	-
Type 1 diabetes	102	34
Type 2 diabetes	136	45
Not sure	62	21
Duration of diabetes	-	-
Less than 1 year	97	32
1-5 years	108	36
6-10 years	70	23
More than 10 years	25	8
Current treatment	-	-
Oral medications	100	33
Insulin injections	114	38
Both	54	18
None	32	11
Do you have any of the following diabetes-related complications?	-	-
Diabetic retinopathy (eye issues)	131	44
Diabetic nephropathy (kidney issues)	120	40
Diabetic foot/ulcers	32	11
Hypertension (high blood pressure)	12	4
None of these	5	2
Smoking	-	-
Used to, but quit	30	10
No	56	19
Yes	214	71
Family history	-	-
Not sure	20	7
No	60	20
Yes	220	73

Table 2 indicates that neuropathic symptoms were relatively common among the 300 participants. Based on the SFN-SIQ with a cut-off score of ≥6, an estimated 37-50% of participants (approximately 111-150 individuals) likely had probable SFN. Similarly, using the DN4 with a threshold of ≥4, 31-50% of participants (about 93-150 individuals) were estimated to experience neuropathic pain. These findings suggest a substantial prevalence of both SFN and neuropathic pain symptoms in the study population, highlighting the importance of screening for these conditions in clinical settings.

**Table 2:** Estimated prevalence of neuropathic symptoms (N = 300) Based on established diagnostic thresholds (SFN-SIQ ≥6; DN4 ≥4), an estimated 37–50% of participants met criteria for probable small fiber neuropathy, while 31–50% met criteria for neuropathic pain.

Scale	Cut-off Criterion	Estimated Prevalence (%)	Approximate Cases (n)
Small Fiber Neuropathy Symptoms Inventory Questionnaire (SFN-SIQ)	≥6	37–50%	111–150
Douleur Neuropathique Questionnaire (DN4)	≥4	31–50%	93–150

Table 3 presents the Spearman correlation coefficients of the very important factors in their study of 300 persons (N = 300). Both SFN-SIQ and DN4 symptoms showed significant positive correlations with all clinical variables. The type, duration of diabetes, current treatment, diabetes related complications, smoking status and family history of diabetes were positively associated with SFN-SIQ scores (type of diabetes, r = 0.26, p < 0.01; duration of diabetes, r = 0.27, p < 0.01; current treatment, r = 0.25, p < 0.01; diabetes-related complications, r = 0.23, p < 0.01; smoking status, r = 0.24, p < Next, DN4 scores were the most strongly correlated with current treatment (r = 0.28, p < 0.01) and were also related to SFN-SIQ scores (r = 0.29, p < 0.01), implying that there is a tight connection between SFN and neuropathic pain-related symptoms. There were significant statistical correlations between all the relationships, meaning that the greater the symptom burden, the more durable the disease, and complications and associated behavioural or familial risk factors are more likely.

**Table 3:** Spearman's correlations among study variables (N = 300) Values represent Spearman's rank-order correlation coefficients (ρ) with corresponding p-values (N = 300), degrees of freedom = 298, *: p < 0.05 considered significant; **: p < 0.01 considered highly significant. Small Fiber Neuropathy Symptoms Inventory Questionnaire (SFN-SIQ) [12], Douleur Neuropathique Questionnaire (DN4) [13].

Variable	1	2	3	4	5	6	7
Type of diabetes	–	–	–	–	–	–	–
Duration of diabetes	ρ = 0.19**, t = 3.36, p = 0.001	–	–	–	–	–	–
Current treatment	ρ = 0.23**, t = 4.07, p <0.001	ρ = 0.22**, t = 3.90, p <0.001	–	–	–	–	–
Diabetes-related complications	ρ = 0.21**, t = 3.72, p <0.001	ρ = 0.18**, t = 3.08, p =0.002	ρ = 0.20**, t = 3.55, p <0.001	–	–	–	–
Smoking status	ρ = 0.17**, t = 2.96, p =0.003	ρ = 0.19*, t = 2.63, p =0.009	ρ = 0.18**, t = 3.12, p =0.002	ρ = 0.16**, t = 2.78, p =0.006	–	–	–
Family history of diabetes	ρ = 0.20**, t = 3.55, p <0.001	ρ = 0.19**, t = 3.31, p =0.001	ρ = 0.21**, t = 3.73, p <0.001	ρ = 0.19**, t = 3.31, p =0.001	ρ = 0.22**, t = 3.85, p <0.001	–	–
Small Fiber Neuropathy Symptoms Inventory Questionnaire [12]	ρ = 0.26**, t = 4.65, p <0.001	ρ = 0.27**, t = 4.84, p <0.001	ρ = 0.25**, t = 4.47, p <0.001	ρ = 0.23**, t = 4.11, p <0.001	ρ = 0.24**, t = 4.28, p <0.001	ρ = 0.27**, t = 4.84, p <0.001	–
Douleur Neuropathique Questionnaire [13]	ρ = 0.24**, t = 4.28, p <0.001	ρ = 0.21**, t = 3.73, p <0.001	ρ = 0.28**, t = 5.02, p <0.001	ρ = 0.22**, t = 3.90, p <0.001	ρ = 0.20**, t = 3.55, p <0.001	ρ = 0.25**, t = 4.47, p <0.001	ρ = 0.29**, t = 5.21, p <0.001

Table 4 presents the findings of the Mann-Whitney U test comparing male and female groups for different diabetes variables. Gender variations were significant on all the variables that were measured. The median score as regards type of diabetes (U = 3672.00, p = 0.013, r = 0.14), duration of diabetes (U = 3578.00, p = 0.006, r = 0.16), and current treatment (U = 3421.00, p = 0.002, r = 0.18) was higher in female participants compared to the male participants. Also, they indicated much higher scores in diabetes-related complications (U = 3698.00, p = 0.016, r = 0.14) and their smoking status (U = 3369.00, p = 0.001, r = 0.19). Additionally, females showed an increased score in three points, namely family history of diabetes (U = 3301.00, p < 0.001, r = 0.20), SFN symptoms (SFN-SIQ) (U = 3514.00, p = 0.004, r = 0.17), as well as neuropathic pain symptoms (DN4) (U = 3436.00, p = 0.003, r = 0.17. Even though all effect sizes fell within a small range (r = 0.14 to 0.20), the overall significance of the correlation between variables gives a hint that female participants could be more affected by diabetes-related symptoms and complications.

**Table 4:** Mann–Whitney U test comparing males and females on diabetes-related variables (N = 300) N: number of participants; the Mann-Whitney U test was used to compare male and female participants across multiple diabetes-related variables; r is the effect size calculated as Z/√N, where N = 300. Small Fiber Neuropathy Symptoms Inventory Questionnaire (SFN-SIQ) [12], Douleur Neuropathique Questionnaire (DN4) [13]. All comparisons show statistically significant gender differences. Effect size interpretations follow Cohen's (1988) guidelines: 0.10 is considered a small effect, 0.30 is considered a medium effect, and 0.50 is considered a large effect. *: p < 0.05, **: p < 0.01 considered significant.

Variable	Male (N = 266); median	Female (N = 34); median	U	Z	P	r
Type of diabetes	148.10	174.26	3672.00	-2.47	0.013^*^	0.14
Duration of diabetes	146.55	179.65	3578.00	-2.74	0.006^**^	0.16
Current treatment	145.02	186.91	3421.00	-3.13	0.002^**^	0.18
Diabetes-related complications	147.43	172.24	3698.00	-2.40	0.016^*^	0.14
Smoking status	144.96	188.12	3369.00	-3.28	0.001^**^	0.19
Family history of diabetes	143.76	190.68	3301.00	-3.51	<0.001^**^	0.20
Small Fiber Neuropathy Symptoms Inventory Questionnaire (SFN-SIQ) [12]	146.05	181.26	3514.00	-2.87	0.004^**^	0.17
Douleur Neuropathique Questionnaire (DN4) [13]	143.88	185.35	3436.00	-2.92	0.003^**^	0.17

Table 5 presents the findings of the Kruskal-Wallis H test used to compare the means of scores on the SFN-SIQ and the DN4 among 300 participants (N = 300) across three groups, which were categorized by type of diabetes. There was a significant variation between the groups, as indicated by the analysis. The Kruskal-Wallis test was substantial (χ²(2) = 11.54, p = 0.003) regarding SFN-SIQ scores, with individuals who were uncertain about their type of diabetes (n = 62) showing significantly lower average ranks (108.94) than those with type 1 (163.25) and type 2 diabetes (159.88), respectively. Likewise, the DN4 scores varied considerably by type of diabetes (χ²(2) = 9.96, p = 0.007). Again, the lowest mean ranks (114.05) did not belong to a sound-minded individual who was unsure whether they had diabetes or not, but to a patient with type 1 or type 2 diabetes. These findings imply that patients with a known diagnosis (type 1 or 2) had higher levels of symptom burden. In contrast, patients unsure of their condition may have reported fewer neuropathic symptoms in part because they were less aware or underdiagnosed.

**Table 5:** Kruskal–Wallis test results for Small Fiber Neuropathy Symptoms Inventory Questionnaire and Douleur Neuropathique Questionnaire by type of diabetes (N = 300) N: number of participants; χ²: effect size; Kruskal–Wallis H test used; **: p < 0.01 considered significant. Small Fiber Neuropathy Symptoms Inventory Questionnaire (SFN-SIQ) [12], Douleur Neuropathique Questionnaire (DN4) [13].

Variable	Type of Diabetes	N	Mean Rank	χ²(2)	p
Small Fiber Neuropathy Symptoms Inventory Questionnaire (SFN-SIQ) [12]	Type 1 diabetes	102	163.25	-	-
-	Type 2 diabetes	136	159.88	-	-
-	Not sure	62	108.94	-	-
Total	-	300	-	11.54	0.003^**^
Douleur Neuropathique Questionnaire (DN4) [13]	Type 1 diabetes	102	160.72	-	-
-	Type 2 diabetes	136	157.48	-	-
-	Not sure	62	114.05	-	-
Total	-	300	-	9.96	0.007^**^

Table 6 presents the Kruskal-Wallis H test results for SFN (SFN-SIQ) and neuropathic pain symptoms (DN4) across four treatment groups. This finding was statistically significant, indicating differences in symptom scores based on the current treatment for diabetes. In the case of SFN-SIQ, the test was also substantial (χ²(3) = 13.42, p = 0.004). The results show that the mean rank is lowest (103.11) among patients in the treatment group of none when compared with the oral medication treatment group (161.92), insulin injection treatment group (158.36), and both treatments (152.87). Again, in DN4 scores, the Kruskal-Wallis statistics were also significant (χ² (3) = 15.87, p = 0.001), with the untreated group once again recording the lowest mean rank (102.94). These results indicate that patients receiving pharmacological treatments (oral medications, insulin, or both) reported higher symptom scores than those who reported receiving no treatment. The lower scores observed in the untreated group should be interpreted with caution, as this may reflect underdiagnosis, differences in disease awareness, or reporting bias rather than a true absence of symptoms.

**Table 6:** Kruskal–Wallis test results for Small Fiber Neuropathy Symptoms Inventory Questionnaire and Douleur Neuropathique Questionnaire by current diabetes treatment (N = 300) N: number of participants; χ²: effect size; Kruskal–Wallis H test used; **: p<0.01 considered significant. Small Fiber Neuropathy Symptoms Inventory Questionnaire (SFN-SIQ) [12], Douleur Neuropathique Questionnaire (DN4) [13].

Variable	Current Treatment	n	Mean Rank	χ²(3)	p
Small Fiber Neuropathy Symptoms Inventory Questionnaire (SFN-SIQ) [12]	Oral medications	100	161.92	-	-
-	Insulin injections	114	158.36	-	-
-	Both	54	152.87	-	-
-	None	32	103.11	-	-
Total	-	300	-	13.42	0.004^**^
Douleur Neuropathique Questionnaire (DN4) [13]	Oral medications	100	164.88	-	-
-	Insulin injections	114	157.02	-	-
-	Both	54	160.15	-	-
-	None	32	102.94	-	-
Total	-	300	-	15.87	0.001^**^

Table 7 presents two multiple regression models examining the effects of factors contributing to diabetes on SFN (SFN-SIQ) and neuropathic pain symptoms (DN4). In both outcomes, the predictors were all positively related, and their indicators were all statistically significant (p < 0.01). Family history (B = 14.22, 0.108, p = 0.002) and smoking (B = 12.35, 0.102, p = 0.003) were the most prominent predictors of SFN-SIQ scores with diabetes-related complications (B = 10.66, 0.116, p = 0.001), and other aspects like type, duration, and treatment also came into play. The same applies to DN4 scores, where family history (B = 10.11, 0.166, p = 0.002) and current treatment (B = 7.40, 0.172, p = 0.003) were the most significant factors. At the same time, duration, complications, and smoking continued to have a positive impact. Our findings demonstrate that increased burden of the SFN and neuropathic pain symptoms is observed in patients with longer disease duration, continual treatment process, and complications, having a level of smoking, and family history of diabetes.

**Table 7:** Multiple linear regression predicting small fiber neuropathy and neuropathic pain (DN4) scores from diabetes-related factors (N = 300) B: unstandardized regression coefficient; SE: standard error; β: standardized regression coefficient; CI: confidence interval; LL: lower limit; UL: upper limit; All predictors were positively correlated with the outcome variables; p < 0.01 denoted as 0.001, 0.004, etc; two-tailed significance testing was used; **: p < 0.01 considered significant.

Predictor	B	SE	β	t	p	95%CI LL	UL
Outcome:(rank of small fiber neuropathy)	122.315	25.812	—	4.74	<0.001**	71.72	172.91
Type of diabetes	11.423	3.892	0.105	2.94	0.004**	3.76	19.08
Duration of diabetes	7.851	2.734	0.093	2.87	0.005**	2.47	13.23
Current treatment	8.267	2.948	0.101	2.80	0.006**	2.47	14.07
Diabetes-related complications	10.664	3.229	0.116	3.30	0.001**	4.31	17.02
Smoke	12.354	4.097	0.102	3.02	0.003**	4.33	20.38
Family history	14.219	4.648	0.108	3.06	0.002**	5.07	23.37
Outcome:(rank of DN4)	132.482	16.143	—	8.21	<0.001**	100.69	164.27
Type of diabetes	8.746	2.951	0.162	2.96	0.004**	2.95	14.54
Duration of diabetes	6.123	2.018	0.148	3.03	0.003**	2.16	10.09
Current treatment	7.398	2.489	0.172	2.97	0.003**	2.50	12.29
Diabetes-related complications	6.215	2.366	0.145	2.63	0.009**	1.56	10.87
Smoke	9.038	2.976	0.152	3.04	0.003**	3.18	14.90
Family history	10.106	3.235	0.166	3.13	0.002**	3.75	16.46

Table 8 presents the findings of chi-square tests to determine the relationship between age category and two variables related to diabetes: the type of diabetes and the family history of diabetes. It was noted that age and the type of diabetes showed a significant association (χ²(8) = 24.9, p = 0.001), with the maximum frequencies of both types of diabetes occurring in individuals more than 60 years old. Again, there was a strong correlation between age and family history of diabetes (χ²(8) = 32.3, p < 0.001), with the group above 60 years recording the highest percentage of positive family histories. These findings indicate that the odds of a known type of diabetes, as well as reporting family history, increase with an increase in age, which would be representative of cumulative risk exposure and later in age being diagnosed.

**Table 8:** Association of age with type of diabetes and family history of diabetes (N = 300) %: percentage; df: degree of freedom; x2: effect size; p: level of significance; p-values calculated using the chi-square test; the significance level is set at p < 0.05; older age groups (especially above 60 years) showed higher prevalence across all categories; all expected cell counts were ≥5, meeting assumptions of the chi-square test; **: p < 0.01 considered significant.

Age	Type 1 Diabetes	Type 2 Diabetes	Not Sure	Total	df	p	x^2^
-	-	-	-	-	8	0.001^**^	24.9
18–30 years	6	5	4	15	-	-	-
31–40 years	14	16	9	39	-	-	-
41–50 years	22	30	11	63	-	-	-
51–60 years	24	35	18	77	-	-	-
Above 60 years	36	50	20	106	-	-	-
Total	102	136	62	300	-	-	-
Age	Not Sure	No	Yes	Total	df	p	x^2^
-	-	-	-	-	8	<0.001^**^	32.3
18–30 years	2	3	10	15	-	-	-
31–40 years	4	6	12	22	-	-	-
41–50 years	5	10	25	40	-	-	-
51–60 years	6	16	50	72	-	-	-
Above 60 years	13	25	123	161	-	-	-
Total	30	60	220	300	-	-	-

## Discussion

The objective of the study was to determine the prevalence and symptom burden of SFN in patients with diabetes, as assessed by the SFN-SIQ, and the presence of neuropathic pain symptoms, as measured by the DN4 questionnaire. The results indicated a significant amount of SFN-related symptom burden in patients with diabetes, demonstrating definite links between neuropathic symptoms and a variety of clinical and demographic findings. The association between the type and duration of diabetes and increased SFN symptom burden also showed significance (r = 0.26 and r = 0.27, respectively; p < 0.01), which demonstrates that a more severe and chronic type of diabetes is associated with increased nerve dysfunction [3]. The higher symptom burden among patients on treatment may reflect greater illness severity; however, this interpretation should be considered with caution, as causality cannot be confirmed [15]. The relationship between complications and diabetes was strongly associated with greater intensity of SFN symptoms, as there is supporting literature where it has been demonstrated that minor fiber damage contributes to diabetes complications, including foot ulcers, painful neuropathy, and autonomic dysfunction [16]. There was a positive association between smoking and the SFN symptoms, which is in line with earlier arguments indicating that smoking could be a predisposing risk of impaired minor nerve fiber damage [17]. The presence of a positive family history of diabetes was found to correspond to increased SFN symptom burden as well, which confirms genetic investigations where the inherited predisposition is recognized as one of the determinants of diabetic neuropathy [18].

A worsening of neuropathic pain was also seen, relating to type 2 diabetes patients, concordant with prior research findings that type 2 diabetes patients experience worse chronic pain and neuropathy [19]. The length of diabetes was positively related to DN4 scores, in line with previous studies that reported duration as an influential risk factor regarding painful diabetic neuropathy [20]. Patients receiving active treatments reported higher neuropathic pain scores, which could suggest more severe disease [21]; however, this interpretation remains tentative given the cross-sectional design. Complications of diabetes are associated with the increased prevalence of neuropathic pain symptoms, as it was found in the studies that the patients who had retinopathy experienced a higher onset of peripheral neuropathy [22]. Differences in the smoking status were again associated with a higher DN4 score, and this aspect has been previously found by other studies that have addressed it as a predictor of painful diabetic neuropathy [23]. The presence of a family history of diabetes was also correlated with the symptoms of nerve pain, as proven by the research that reported the increased prevalence of neuropathy in people with diabetic relatives [24]. In our study, SFN-SIQ and DN4 scores showed a significant correlation, indicating that small fiber syndrome exhibits symptoms that closely reflect neuropathic pain. This aligns with prior studies, including a clinical trial where 90% of patients with diabetes with neuropathic pain displayed unquestionable small-fiber damage, indicating that SFN and pain are closely connected even in the absence of considerable fiber loss [25].

Female participants in our study possessed a slightly worse type of diabetes, along with a greater disease duration. This is consistent with data obtained in recent literature with the focus on sex-based disparities in diabetes recognition and development, where women have a delayed identification and peculiar biological and social determinants that increase the severity and chronicity of illness [26]. We found that women had an increased likelihood of being underactive in treatment, including insulin. Although other studies indicate that women change therapies more regularly, men might be more likely to undergo insulin-based intensification at higher levels of glycemia. Such results emphasize the difference in responses to treatment and clinical decision-making dependent on sex [27]. Females in our study reported a higher prevalence of complications of diabetes, which include neuropathy and vision problems. However, this observation is not supported by studies, where it was concluded that males were affected statistically significantly more by neuropathy, retinopathy, nephropathy, and cardiovascular disease. The difference between the estimated figures can indicate sample peculiarities or unquantified confounding factors in our analysis [28]. Most people report that smoking is more common among men, but our research found a much greater smoking-symptoms-related burden among our female participants. This is in line with recent reports that indicate that women with diabetes are especially susceptible to vascular and neuropathic outcomes of smoking. They find it harder to quit, and they are liable to smoke-related complications [29]. Our findings indicated that a family history of diabetes was more frequent in females; however, earlier studies also suggest that the genetic and familial contribution to type 2 diabetes can be higher in males, especially in individuals with early-onset type 2 diabetes. This difference may be due to sampling or variance, or it may be due to recall/reporting bias in our population [30]. In our study, female participants appeared to report more symptoms of SFN than males. This observation is partly consistent with previous studies suggesting that female sex may be associated with a greater risk of painful diabetic peripheral neuropathy, possibly due to differences in pain perception and pathophysiology [31]. However, this finding should be interpreted cautiously, given the small number of female respondents in our sample. Our results that participants with female sex showed greater frequency of nerve pain indicators, as measured by DN4, align with previous evidence that women report both increased frequency and intensity of neuropathic pain despite objective nerve damage being less severe [32].

Participants with a known diagnosis of type 1 and type 2 diabetes reported more symptoms of SFN (SFN-SIQ) and nerve pain (DN4) compared to those who did not know the type of diabetes. These results are consistent with a longitudinal trial, which demonstrated that patients with confirmed type 1 and type 2 diabetes, and particularly type 2 patients, experienced increased minor fiber damage and neuropathic symptoms with time [33].

In our study, patients who were undergoing pharmacological treatment of diabetes, especially with oral agents and insulin, were found to express far more symptoms of SFN and nerve pain. The finding is in line with the findings of the past, where it was pointed out that an abrupt glycemic control in treated patients could precipitate treatment-induced neuropathy, particularly in painful small fiber cases [34]. Overall, diabetes type and duration, presence of complications, smoking, and positive family history were significantly associated with symptom burden, supporting their potential role in the risk profile of SFN and diabetic neuropathic pain [3,15-24].

In our study, instances of both type 1 and type 2 diabetes were most frequently recorded among respondents who were over 60 years old. This is in line with estimates of the global proportion of people living with diabetes, which have shown that almost one-fifth of the population aged 65 years and above are living with diabetes, a factor that illustrates the prevalence of diabetes in older people [35]. Furthermore, in our research, the risk of a positive family history of diabetes was increased in older people and more specifically in those over 60. This is in line with evidence, which showed that a family history was found to be a strong contributor to the development of diabetes in people over the age of 45 years, particularly when interacting with other diseases such as hypertension [36].

The study as a whole demonstrates the validity of using SFN-SIQ as a reliable method for establishing symptom burden in general diabetic management. It highlights the need to incorporate neuropathic symptom assessment into regular clinical practice.

Limitations

There are several limitations associated with this study, despite the strengths. To begin with, the study employed a cross-sectional design, which is not suitable for determining a causal relationship between diabetes-related factors and symptom burden of SFN. They would require longitudinal studies to analyze the development of symptoms. Second, sampling in the hospital environment may limit the ability to generalize the studied results to a broader population of diabetics, particularly in rural or underserved regions. Moreover, the relatively small sample size of 300 patients may also restrict the generalizability of the findings, and larger multicenter studies are warranted. Third, the SFN-SIQ and DN4 are also validated; however, they have the issue of being self-reports, which are susceptible to recall bias or subjective interpretation. The limited representation of females, together with the predominance of male and smoker participants, may restrict the generalizability of our findings, as the symptom patterns observed in this study may not fully capture the experiences of women and non-smokers with diabetes. Furthermore, only the interview portion of the DN4 was used, excluding the physical examination component that could have strengthened diagnostic accuracy. Most importantly, no additional clinical investigations (such as neurological examination, QST, or skin biopsy) were performed, which may have reduced diagnostic precision. In addition, detailed data regarding participants' medication use were not collected, and long-term glycemic control indicators (such as HbA1c) were not assessed, which limited our ability to evaluate their contribution to SFN symptom burden. Future studies should incorporate longitudinal designs, community-based sampling, objective clinical assessments, and detailed data on medication and glycemic control to validate and extend these findings.

Future directions

Future studies may benefit from adopting a longitudinal design that allows for the comparison of how SFN symptoms evolve across different treatment adherence, glycemic control, and comorbidities. Additionally, measures of objective diagnosis, such as skin biopsies or QST, should be introduced to provide validation for self-reported symptom burden. The representation of gender should also be made more equitable; in particular, more females should be included to better define sex-specific differences in the frequency and manifestations of minor fiber neuropathy symptoms. Studies of more comprehensive and diverse populations (reaching people in rural and less-served areas) would yield more generalizable results. Furthermore, future research can also enhance the quality of specific, targeted interventions, such as lifestyle changes or certain pharmacological substances, to alleviate the symptom burden associated with SFN.

## Conclusions

This study underscores a significant rate of minor fiber neuropathy symptoms and neuropathic pain in patients with diabetes. Significant correlations were observed between symptom burden and clinical variables, including the length of diabetes, the current regimen of treatment, complications, smoking, and family history. Future studies focused on developing screening and diagnostic tools in outpatient diabetic clinics should consider the SFN-SIQ and DN4 as effective methods of screening in such a scenario, since they can also be used as routine clinical assessment tools. These results underscore the importance of screening for SFN in patients with diabetes and emphasize the value of early recognition as part of clinical care. However, further longitudinal studies are necessary to confirm causal relationships. Increased knowledge among clinicians, with the availability and more straightforward application of screening tools, may contribute to earlier diagnosis, symptom improvement, and ultimately, an improved quality of life for patients with diabetes.

## Figures and Tables

**Figure 1:**
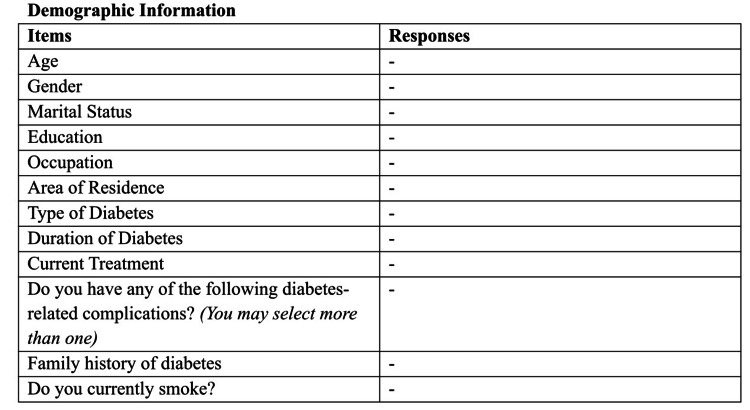
Questionnaire

## Appendices

Appendix 1
